# Supplementary material for: A computational text analysis investigation of the relation between personal and linguistic agency
Source: Commun Psychol. 2023 Sep 25;1:23. doi: 10.1038/s44271-023-00020-1 (PMC11332215; doi:10.1038/s44271-023-00020-1)
Supplement: Supplementary file 1 — Supplementary Information [file 44271_2023_20_MOESM1_ESM.pdf]

Supplementary Information: A Computational Text Analysis Investigation of the Relation Between Personal and Linguistic Agency

Supplementary Methods

Supplementary Notes 1-3

Supplementary Figures S1-S2

Supplementary Tables S1-S12

Supplementary Methods

Messages on Twitter differ in their target audience. Top-level messages (i.e., “posts”) target the entire social network of a single user. However, a conversation produces lower-levels messages (i.e., “replies”) that usually come in response to a post or to another comment in the conversational chain. Is there a difference in the relationship between passive voice and followership, as a function of message level?

To examine the relationship between passive voice usage and message level, we used the same random sample from the CCR analyses with a total of N = 100,000 posts. After excluding multiple posts by individual users, the sample size was reduced to N = 81,606. In our analysis, we investigated how the use of passive voice varied based on the message level (i.e., top-level vs. replies), controlling for tweet length (median center). We found that the main effect of passive voice remained significant and exhibited the same direction (IRR = 0.82, 95% CI [0.77, 0.88], $p < .001$) and there was a main effect of tweet length (IRR = 1.05, 95% CI [1.05, 1.05], $p < .001$). Additionally, we observed a main effect of message level, indicating that replies were associated with users who had fewer followers (IRR = 0.50, 95% CI [0.48, 0.51], $p < .001$). However, we did not find evidence of an interaction between passive voice usage and response level (IRR = 0.93, 95% CI [0.84, 1.03], $p = 0.17$).

Supplementary Notes

1. Annotation instructions:

We are interested in **non-agentive** language.

Agentive language refers to the case where a sentence has a specified *agent*. **Someone** who is **responsible** for the event. For example: *Adam kicked the ball*. In This case, Adam is the agent as he kicked the ball. In the sentence “the ball was kicked” or “the ball got kicked” there is no agent – the ball was just “kicked” by an unknown person. However, when a passive sentence followed by a “by” phrase (e.g., “the ball got kicked by Adam”) it **is** considered an agentive sentence. Therefore, not all passive sentences are considered non-agentive.

<u>More examples for non-agentive sentences:</u>

“The vase broke” (vs. “John broke the vase”)

“The book was put on the table” (vs. “Michelle put the book on the table”)

“The curtain caught fire” (vs. “I set the curtain on fire”)

Your job is to identify instances of non-agentive language and count them in the given texts. For each text, please write down the number of non-agentive instances in the associated text.

<u>Examples:</u>

1) “Michael went to get some beer. He drove his car into the nearest “Shufersal” and parked his car. **The car bumped into an e-scooter**, knocking over the e-scooter driver **who had to be taken to nearest hospital**” (count = 2)
2) “Michael apologized to the e-scooter driver and promised to pay his medical bills” (count = 0)

2. List of 100 random subreddits out of the 1000 popular subreddits:
   whatisthisthing, bipolar, lewronggeneration, wicked_edge, FlashTV, oddlysatisfying, islam, Boxing, hcteams, dogecoin, offbeat, OkCupid, AskUK, dota2loungebets, shittyfoodporn, steroids, Psychonaut, europe, Muse, sysadmin, montreal, toronto, fitnesscirclejerk, poker, DestinyTheGame, TokyoGhoul, DotA2, neopets, gameswap, portugal, AskDocs, AndroidGaming, wsgy, airsoft, Frugal, ClashOfClans, chelseafc, AsiansGoneWild, washingtondc, jailbreak, Wet_Shavers, punchablefaces, asoiaf, opiates, GrandTheftAutoV, pittsburgh, UkrainianConflict, adventuretime, vinyl, PetiteGoneWild, pcgaming, CatsStandingUp, self, HeistTeams, GameSale, crossfit, BasicIncome, spikes, Catholicism, excel, relationships, running, Spiderman, baseball, weddingplanning, MTB, Firearms, ecigclassifieds, AskMen, Unity3D, Surface, OnePieceTC, CrazyIdeas, tipofmytongue, OutOfTheLoop, howardstern, TopGear, TheDescendantsOfRome, frugalmalefashion, cordcutters, ravens, AndroidQuestions, RiotFreeLoL, DFO, linux, exjw, Pathfinder_RPG, Bad_Cop_No_Donut, Suomi, onewordeach, eu4, badhistory, TrollXChromosomes, nsfw, smashbros, madmen, EDH, NYYankees, askcarsales, offmychest.

3. List of “support groups” subreddits:
   AskDocs, LegalAdvice, PersonalFinance, Cooking, HomeImprovement, Fitness, Gardening, DIY, Travel, Pets, Parenting, Entrepreneur, Technology, Health, AskScience, AskHistorians, AskEngineers, AskCulinary, AskPhotography, AskWomen, AskMen, AskProgramming, AskEconomics, AskMarketing, AskPhysics, AskStatistics, AskAnthropology, AskSocialScience, AskComputerScience, AskPhilosophy, AskSociology, AskScienceFiction, AskGIS, AskHR, AskVet, AskElectronics, AskLawyers, AskEngineers, AskEconomists, AskAstronomy, AskBiology, AskChemistry, AskGeology, AskHistory, AskLinguistics, AskLiteraryStudies, AskMath, AskNeuroscience, AskPhysics, AskPoliticalScience, AskScienceDiscussion, AskSocialWork, AskStatistics, AskTeachers, AskUK, AskWomenOver30, AskMenOver30, AskWomenAdvice, AskMenAdvice, AskFeminists, AskReligion, AskDIY, AskPhotography, AskVideography, AskFitness, AskRunning, AskCycling, AskHiking, AskGardening, AskLandscaping, AskArchery, AskFishing, AskBoating, AskSailing, AskHomebrewing, AskCocktails, AskWine, AskArt, AskDesign, AskWriting, AskMusic, AskFilm, AskTheatre, AskCrafts, AskGameDev, AskWebDesign, AskGraphicDesign, AskMarketing, AskEntrepreneurship, AskInvesting, AskStockMarket, AskRealEstate, AskFinance, AskBusiness.

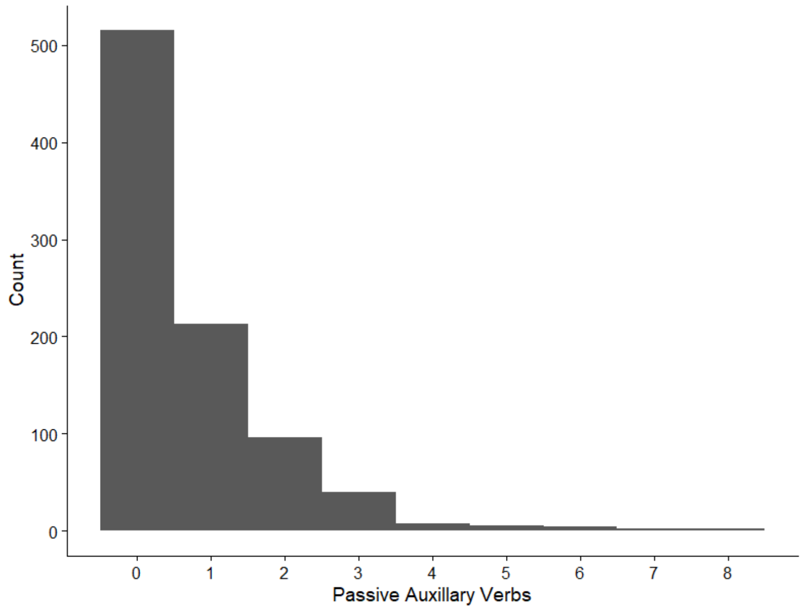

Figure S1. Histogram of passive auxiliary verbs in Study 1.

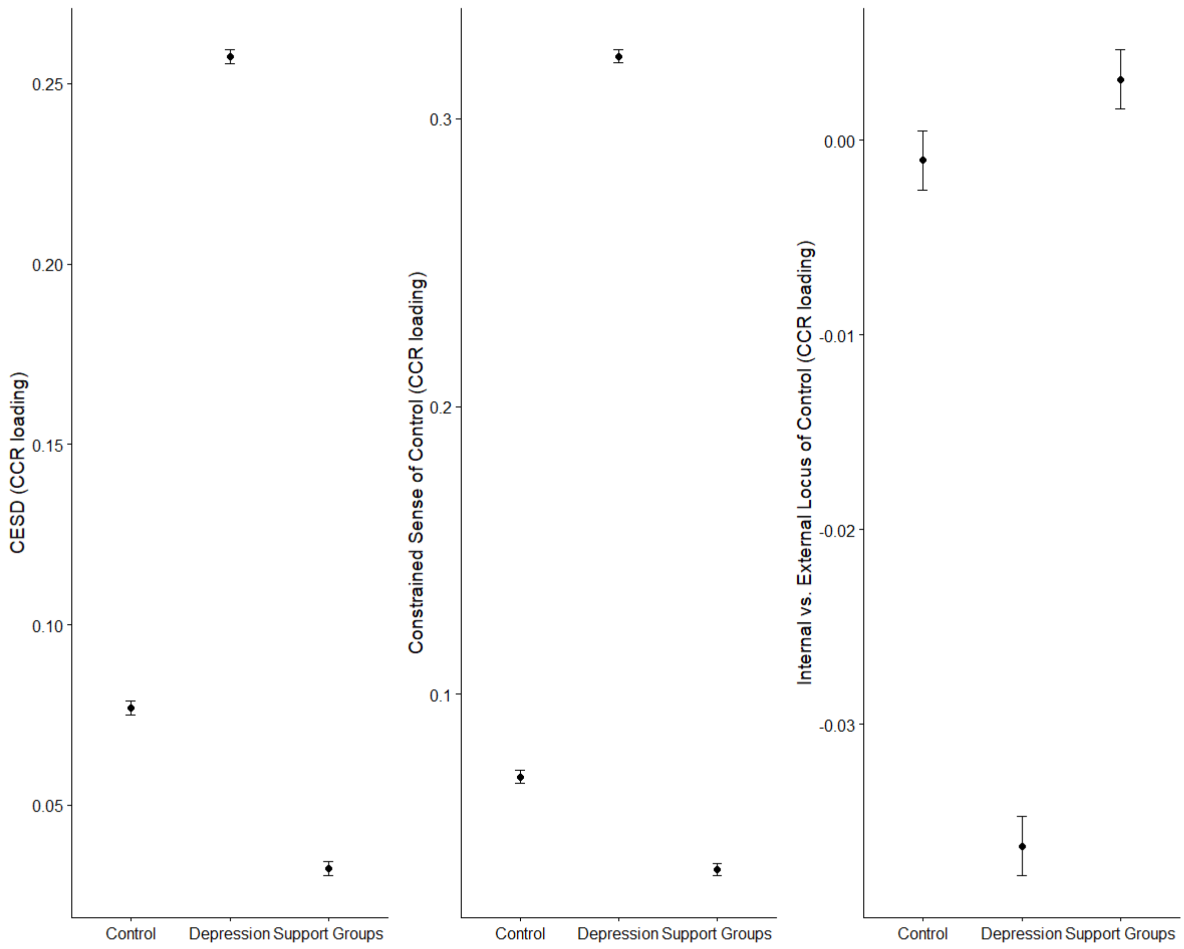

Figure S2. Study 3c: CCR loadings by group (Reddit community) and construct of interest (CESD: depression; constrained sense of control; locus of control). Dots denote average CCR loadings (cosine similarity), error bars denote 95% confidence intervals.

Table S1. Negative Binomial Generalized Linear Model predicting passive auxiliary verbs as a function of power condition (high vs. low) and self-referential language (I-words) in Study 1. Self-referential language and word count are median-centered.

	**Passive Count**				
Predictors	*Incidence Rate Ratios*	*std. Error*	*CI*	*Statistic*	*p*
(Intercept)	0.46	0.04	0.38 – 0.54	-8.92	**<0.001**
Condition [Low Power]	1.65	0.17	1.35 – 2.02	4.86	**<0.001**
I-words	0.96	0.02	0.92 – 1.01	-1.74	0.082
Group [prolific]	0.94	0.09	0.78 – 1.13	-0.68	0.498
Word Count	1.02	0.00	1.01 – 1.02	10.66	**<0.001**
Condition [Low Power] × I-words	0.97	0.02	0.94 – 1.01	-1.55	0.120
Observations	835				

Table S2. Negative Binomial Generalized Linear Model predicting passive auxiliary verbs as a function of power condition (high vs. low) and self-referential language (I-words) in Study 1, controlling for gender. Self-referential language and word count are median-centered.

	passive_count				
Predictors	*Incidence Rate Ratios*	*std. Error*	*CI*	*Statistic*	*p*
(Intercept)	0.47	0.08	0.33 – 0.66	-4.22	**<0.001**
Condition [Low Power]	1.56	0.17	1.27 – 1.92	4.20	**<0.001**
I-words	0.96	0.02	0.92 – 1.00	-2.03	**0.042**
Group [prolific]	0.93	0.09	0.77 – 1.13	-0.72	0.469
Word Count	1.02	0.00	1.01 – 1.02	10.33	**<0.001**
Gender (women)	1.03	0.10	0.85 – 1.24	0.28	0.781
Condition [Low Power] × I-words	0.98	0.02	0.95 – 1.02	-0.90	0.371
Observations	785				
R^2 Nagelkerke	0.339				

Table S3. Negative Binomial Generalized Linear Model predicting number of I-words (self-referential language) as a function of number of group condition in Study 1. Word count are median-centered.

	**I-words**				
Predictors	*Incidence Rate Ratios*	*std. Error*	*CI*	*Statistic*	*p*
(Intercept)	5.90	0.15	5.61 – 6.20	70.33	**<0.001**
Condition [Low Power]	1.29	0.04	1.22 – 1.36	8.91	**<0.001**
Group [prolific]	0.89	0.03	0.84 – 0.94	-3.99	**<0.001**
Word Count	1.01	0.00	1.01 – 1.01	36.48	**<0.001**
Observations	835				

Table S4. Negative Binomial Generalized Linear Model predicting number of followers as a function of number of passive auxiliary verbs in tweet and self-referential language (I-words) in Study 2. Self-referential language and word count are median-centered.

	**Followers count**				
Predictors	*Incidence Rate Ratios*	*std. Error*	*CI*	*Statistic*	*p*
(Intercept)	1886.97	2.55	1882.05 – 1891.89	5580.42	**<0.001**
Passive	0.54	0.00	0.53 – 0.55	-72.38	**<0.001**
I-words	0.67	0.00	0.67 – 0.68	-237.32	**<0.001**
Word Count	1.06	0.00	1.05 – 1.06	245.56	**<0.001**
Passive × I-words	1.23	0.01	1.21 – 1.25	27.25	**<0.001**
Observations	2726733				

Table S5. Negative Binomial Generalized Linear Model predicting passive auxiliary verbs as a function of online community (depression vs. random sample of popular communities) and self-referential language (I-words) in Study 3a. Self-referential language and word count are median-centered.

	**Passive Count**				
Predictors	*Incidence Rate Ratios*	*std. Error*	*CI*	*Statistic*	*p*
(Intercept)	0.56	0.02	0.53 – 0.60	-20.24	**<0.001**
Depression	1.26	0.04	1.18 – 1.35	6.67	**<0.001**
I-words	1.00	0.00	1.00 – 1.01	0.54	0.592
Word Count	1.00	0.00	1.00 – 1.00	35.80	**<0.001**
Depression × I-words	0.99	0.00	0.99 – 1.00	-3.31	**0.001**
Observations	8690				

Table S6. Negative Binomial Generalized Linear Model predicting I-words as a function of online community (depression vs. random sample of popular communities) in Study 3a. Word count is median-centered.

	**I-words**				
Predictors	*Incidence Rate Ratios*	*std. Error*	*CI*	*Statistic*	*p*
(Intercept)	4.33	0.06	4.21 – 4.46	98.44	**<0.001**
Depression	2.54	0.04	2.45 – 2.62	52.59	**<0.001**
Word Count	1.00	0.00	1.00 – 1.00	124.95	**<0.001**
Observations	8690				

Table S7. Negative Binomial Generalized Linear Model predicting passive auxiliary verbs as a function of online community (depression vs. random sample of popular communities) and self-referential language (I-words) in Study 3b. Self-referential language and word count are median-centered.

	Passive Count				
Predictors	*Incidence Rate Ratios*	*std. Error*	*CI*	*Statistic*	*p*
(Intercept)	0.47	0.01	0.45 – 0.50	-26.10	**<0.001**
Depression	1.42	0.05	1.32 – 1.52	10.03	**<0.001**
I-words	1.00	0.00	1.00 – 1.00	0.03	0.978
Word Count	1.00	0.00	1.00 – 1.00	39.15	**<0.001**
Depression × I-words	1.00	0.00	0.99 – 1.00	-1.67	0.094
Observations	9685				

Table S8. Negative Binomial Generalized Linear Model predicting I-words as a function of online community (depression vs. random sample of popular communities) in Study 3b. Word count is median-centered.

	**I-words**				
Predictors	*Incidence Rate Ratios*	*std. Error*	*CI*	*Statistic*	*p*
(Intercept)	4.31	0.06	4.19 – 4.43	103.10	**<0.001**
Depression	2.56	0.04	2.48 – 2.65	55.80	**<0.001**
Word Count	1.00	0.00	1.00 – 1.00	136.56	**<0.001**
Observations	9685				

Table S9. Negative Binomial Generalized Linear Model predicting passive auxiliary verbs as a function of online community (depression, support groups and a random sample of popular communities) and self-referential language (I-words) in Study 3c. Self-referential language and word count are median-centered.

	Passive Count				
Predictors	*Incidence Rate Ratios*	*std. Error*	*CI*	*Statistic*	*p*
(Intercept)	0.57	0.01	0.55 – 0.59	-33.28	**<0.001**
Group [Depression]	1.16	0.03	1.11 – 1.22	6.35	**<0.001**
Group [Support Groups]	1.10	0.03	1.06 – 1.16	4.27	**<0.001**
I-words	0.99	0.00	0.99 – 0.99	-11.69	**<0.001**
Word Count	1.00	0.00	1.00 – 1.00	84.62	**<0.001**
Group [Depression] × I-words	1.00	0.00	1.00 – 1.00	1.80	0.072
Group [Support Groups] × I-words	1.01	0.00	1.00 – 1.01	6.15	**<0.001**
Observations	24765				

Table S10. Negative Binomial Generalized Linear Model predicting I-words a function of online community (depression, support groups and a random sample of popular communities) in Study 3c. Word count are median-centered.

	**I-words**				
Predictors	*Incidence Rate Ratios*	*std. Error*	*CI*	*Statistic*	*p*
(Intercept)	5.07	0.05	4.97 – 5.16	174.40	**<0.001**
Group [Depression]	2.06	0.03	2.01 – 2.11	58.01	**<0.001**
Group [Support Groups]	1.07	0.01	1.04 – 1.09	5.09	**<0.001**
Word Count	1.00	0.00	1.00 – 1.00	215.58	**<0.001**
Observations	24765				

Table S11. Negative Binomial Generalized Linear Model predicting passive auxiliary verbs as a function of online community (depression vs. random sample of popular communities) in Study 3a (following pre-registration). Word count is median-centered.

	**Passive Count**				
Predictors	*Incidence Rate Ratios*	*std. Error*	*CI*	*Statistic*	*p*
(Intercept)	0.58	0.02	0.55 – 0.61	-20.15	**<0.001**
Depression	1.19	0.04	1.12 – 1.27	5.38	**<0.001**
Word Count	1.00	0.00	1.00 – 1.00	73.25	**<0.001**
Observations	8690				

Table S12. Negative Binomial Generalized Linear Model predicting passive auxiliary verbs as a function of online community (depression vs. random sample of popular communities) in Study 3b (following pre-registration). Word count is median-centered.

	**Passive Count**				
Predictors	*Incidence Rate Ratios*	*std. Error*	*CI*	*Statistic*	*p*
(Intercept)	0.48	0.01	0.45 – 0.51	-26.26	**<0.001**
Depression	1.38	0.04	1.29 – 1.47	9.87	**<0.001**
Word Count	1.00	0.00	1.00 – 1.00	76.79	**<0.001**
Observations	9685				
