# Supplementary material for: A computational text analysis investigation of the relation between personal and linguistic agency
Source: Commun Psychol. 2023 Sep 25;1:23. doi: 10.1038/s44271-023-00020-1 (PMC11332215; doi:10.1038/s44271-023-00020-1)
Supplement: Supplementary file 3 — Reporting Summary [file 44271_2023_20_MOESM3_ESM.pdf]

nature portfolio

Corresponding author(s): Almog Simchon

Last updated by author(s): Aug 7, 2023

Reporting Summary

Nature Portfolio wishes to improve the reproducibility of the work that we publish. This form provides structure for consistency and transparency in reporting. For further information on Nature Portfolio policies, see our Editorial Policies and the Editorial Policy Checklist.

Statistics

For all statistical analyses, confirm that the following items are present in the figure legend, table legend, main text, or Methods section.

n/a	Confirmed	
☐	☒	The exact sample size (*n*) for each experimental group/condition, given as a discrete number and unit of measurement
☐	☒	A statement on whether measurements were taken from distinct samples or whether the same sample was measured repeatedly
☐	☒	The statistical test(s) used AND whether they are one- or two-sided<br>*Only common tests should be described solely by name; describe more complex techniques in the Methods section.*
☐	☒	A description of all covariates tested
☐	☒	A description of any assumptions or corrections, such as tests of normality and adjustment for multiple comparisons
☐	☒	A full description of the statistical parameters including central tendency (e.g. means) or other basic estimates (e.g. regression coefficient) AND variation (e.g. standard deviation) or associated estimates of uncertainty (e.g. confidence intervals)
☐	☒	For null hypothesis testing, the test statistic (e.g. *F*, *t*, *r*) with confidence intervals, effect sizes, degrees of freedom and *P* value noted<br>*Give P values as exact values whenever suitable.*
☒	☐	For Bayesian analysis, information on the choice of priors and Markov chain Monte Carlo settings
☐	☒	For hierarchical and complex designs, identification of the appropriate level for tests and full reporting of outcomes
☐	☒	Estimates of effect sizes (e.g. Cohen's *d*, Pearson's *r*), indicating how they were calculated

Our web collection on statistics for biologists contains articles on many of the points above.

Software and code

Policy information about availability of computer code

Data collection	R and RStudio,<br>R packages: rtweet, rreddit
Data analysis	R and RStudio,<br>R packages: tidyverse, quanteda, spacyr, emmeans, MASS, broom, cowplot, ngram, text, marginaleffects<br>python packages: spaCy, SentenceTransformers

For manuscripts utilizing custom algorithms or software that are central to the research but not yet described in published literature, software must be made available to editors and reviewers. We strongly encourage code deposition in a community repository (e.g. GitHub). See the Nature Portfolio guidelines for submitting code & software for further information.

Data

Policy information about availability of data

All manuscripts must include a data availability statement. This statement should provide the following information, where applicable:

- Accession codes, unique identifiers, or web links for publicly available datasets
- A description of any restrictions on data availability
- For clinical datasets or third party data, please ensure that the statement adheres to our policy

All sharable data and code are available at https://osf.io/nwsx3

Human research participants

Policy information about studies involving human research participants and Sex and Gender in Research.

Reporting on sex and gender	we used existing data collected by another group, or social media data that does not have any of such characteristics
Population characteristics	we used existing data collected by another group, or social media data that does not have any of such characteristics
Recruitment	we used existing data collected by another group, or social media data where we did not use active participants recruitment
Ethics oversight	Department of Psychology IRB at Ben-Gurion University of the Negev

Note that full information on the approval of the study protocol must also be provided in the manuscript.

Field-specific reporting

Please select the one below that is the best fit for your research. If you are not sure, read the appropriate sections before making your selection.

☐ Life sciences ☒ Behavioural & social sciences ☐ Ecological, evolutionary & environmental sciences

For a reference copy of the document with all sections, see nature.com/documents/nr-reporting-summary-flat.pdf

Behavioural & social sciences study design

All studies must disclose on these points even when the disclosure is negative.

Study description	Quantitative study analyzing text and quantifying the use of passive voice and the relationship to personal agency.
Research sample	Study 1: mturk and prolific participants: See Kasprzyk, L., & Calin-Jageman, R. (2017) https://osf.io/WCH5R/<br>Study 2: Big data Twitter data<br>Study 3: Big data Reddit data
Sampling strategy	Study 1: See Kasprzyk, L., & Calin-Jageman, R. (2017) https://osf.io/WCH5R/<br>Study 2: Tweets were collected from across the United States, including all 50 states and the District of Columbia. We extracted tweets between April 2019 and June 2019.<br>Study 3a: We collected 10,000 messages from the depression subreddit (i.e., Reddit community), and 100 messages from 100 randomly selected subreddits (sampled from a list of 1000 popular subreddit, see SI for the sampled list and online repository for the complete unsampled list). The messages ranged in their time between July 2020 and November 2019.<br>Study 3b: This study was an exact replication of Study 3a, with the exception that the collected data were posted between July 2019 and November 2016. The original sample size consisted of 9,999 messages from the depression subreddit, and 10,001 messages from the control sample. After preprocessing, the final sample size consisted of 9,685 (6,325 from the depression condition).<br>Study 3c: This study was a replication of Studies 3a and 3b, with a different set of control subreddits. To make a comprehensive list of support groups that do not mainly provide emotional support, we prompted ChatGPT4 chatbot (OpenAI, 2023) with the following; “please generate a list of 200 actual reddit forums. make sure to adhere to the following criteria: 1. make sure the forum provides support or assistance. 2. make sure the forum actually exists. 3. make sure the forum doesn't deal with psychological content matter or emotionally-charged topics. 4. make sure that you do not repeat yourself and generate duplicate entries”. This prompt generated 94 subreddits. Final N = 24,765
Data collection	Study 1: See Kasprzyk, L., & Calin-Jageman, R. (2017) https://osf.io/WCH5R/<br>Study 2: rtweet R package through dedicated Twitter API<br>Study 3a-3b: rreddit r package through dedicated Reddit API (Pushshift)<br>Study 3c: The data was obtained by querying a vast dataset of pre-collected Reddit activity (Baumgartner et al., 2020), which is readily accessible on BigQuery
Timing	Study 1: See Kasprzyk, L., & Calin-Jageman, R. (2017) https://osf.io/WCH5R/<br>Study 2: Tweets were collected between April 2019 and June 2019.<br>Study 3a: We collected Reddit data that ranged in their time between July 2020 and November 2019.<br>Study 3b: We collected Reddit data that ranged in their timebetween July 2019 and November 2016.<br>Study 3c: We collected Reddit data that was published in June 2019
Data exclusions	Study 1: responses without text were excluded as they could not be analyzed<br>Study 2: no exclusion<br>Study 3: no exclusion
Non-participation	NA

Randomization	NA

Reporting for specific materials, systems and methods

We require information from authors about some types of materials, experimental systems and methods used in many studies. Here, indicate whether each material, system or method listed is relevant to your study. If you are not sure if a list item applies to your research, read the appropriate section before selecting a response.

Materials & experimental systems

n/a	Involved in the study
☒	☐ Antibodies
☒	☐ Eukaryotic cell lines
☒	☐ Palaeontology and archaeology
☒	☐ Animals and other organisms
☒	☐ Clinical data
☒	☐ Dual use research of concern

Methods

n/a	Involved in the study
☒	☐ ChIP-seq
☒	☐ Flow cytometry
☒	☐ MRI-based neuroimaging
